# Cinnamic Acid, Perillic Acid, and Tryptophan Metabolites Differentially Regulate Ion Transport and Serotonin Metabolism and Signaling in the Mouse Ileum In Vitro

**DOI:** 10.3390/ijms25126694

**Published:** 2024-06-18

**Authors:** Lili Jiang, Youling Hao, Qianjun Li, Zhaolai Dai

**Affiliations:** 1 State Key Laboratory of Animal Nutrition and Feeding, College of Animal Science and Technology, China Agricultural University, Beijing 100193, China; lilijiang2020@163.com (L.J.); hao17863527642@163.com (Y.H.); 2 Laboratory of Animal Genetics, Breeding and Reproduction, College of Animal Science and Technology, China Agricultural University, Beijing 100193, China; lqj1821168324@163.com

**Keywords:** phytochemicals, indoles, serotonin receptors, Ussing chamber, ileum

## Abstract

Phytochemicals and tryptophan (Trp) metabolites have been found to modulate gut function and health. However, whether these metabolites modulate gut ion transport and serotonin (5-HT) metabolism and signaling requires further investigation. The aim of this study was to investigate the effects of selected phytochemicals and Trp metabolites on the ion transport and 5-HT metabolism and signaling in the ileum of mice in vitro using the Ussing chamber technique. During the in vitro incubation, vanillylmandelic acid (VMA) reduced (*p* < 0.05) the short-circuit current, and 100 μM chlorogenic acid (CGA) (*p* = 0.12) and perillic acid (PA) (*p* = 0.14) had a tendency to reduce the short-circuit current of the ileum. Compared with the control, PA and *N*-acetylserotonin treatment upregulated the expression of tryptophan hydroxylase 1 (*Tph1*), while 100 μM cinnamic acid, indolelactic acid (ILA), and 10 μM CGA or indoleacetaldehyde (IAld) treatments downregulated (*p* < 0.05) the mRNA levels of *Tph1*. In addition, 10 μM IAld or 100 μM ILA upregulated (*p* < 0.05) the expression of monoamine oxidase A (*Maoa*). However, 10 μM CGA or 100 μM PA downregulated (*p* < 0.05) *Maoa* expression. All selected phytochemicals and Trp metabolites upregulated (*p* < 0.05) the expression of *Htr4* and *Htr7* compared to that of the control group. VMA and CGA reduced (*p* < 0.05) the ratios of *Htr1a*/*Htr7* and *Htr4*/*Htr7*. These findings may help to elucidate the effects of phytochemicals and Trp metabolites on the regulation of gut ion transport and 5-HT signaling-related gut homeostasis in health and disease.

## 1. Introduction

The gut microbiota, together with its metabolites, has been proven to modulate host metabolism and improve the gut barrier and function [1,2]. Robust gut function is essential for maintaining whole-body nutrition and health. The small intestine is crucial for nutrient absorption and immunity; however, these processes are affected by the gut microbiota, which has attracted much attention in recent years [3]. It has been shown that tryptophan (Trp) metabolites regulate the gut barrier and immune response [4,5]. Indoleacetic acid and indole-3-aldehyde alleviate gut inflammation by decreasing lipopolysaccharide-induced production of the proinflammatory cytokines tumor necrosis factor alpha (TNF-α) and interleukin (IL)-1β in macrophages and promoting the production of IL-22 and the expression of tight junction proteins in the colon of mice [6,7,8]. In addition, a study with aryl hydrocarbon receptor (AHR)^−/−^ mice suggested that indole-3-ethanol and indole-3-pyruvate regulated gut barrier function in an AHR-signaling dependent manner [9]. However, other mechanisms underlying the regulatory role of Trp metabolites in small intestine function require further investigation.

Moreover, phytochemicals, which are naturally bioactive compounds in plants, have attracted great attention because of their health-promoting benefits, including antioxidant, immunomodulatory, and antibacterial activities and the ability to increase the efficiency of nutrient digestion and absorption [10,11,12]. Perillic acid, a major metabolite of D-limonene, regulates the body immune response by increasing the total numbers of white blood cells and promotes the production of the antioxidative compound glutathione in the small intestine of mice [13,14,15]. Curcumin protected against oxidative injury and inflammation of the gut through inhibiting the nuclear factor κ-B (NF-κB)/IκB signaling pathway, decreasing m6A RNA methylation and boosting intestinal autophagy in duckling and piglet models [16,17,18]. In addition, the phenolic compounds cinnamic acid and chlorogenic acid have been proposed to have antioxidant and anti-inflammatory properties [19]. Cinnamic acid can alleviate oxidative stress in the liver by inhibiting macrophage infiltration and downregulating the expression of proinflammatory cytokines such as *TNF-α* and *IL-6* [20,21]. In addition, chlorogenic acid suppressed gut inflammation and reduced hepatotoxicity by downregulating the expression of the proinflammatory cytokines *IL-1β*, *IL-6*, and interferon-γ (*IFN-γ*) and interfering with mitochondrial function and Nrf2/HO-1 signaling [22,23]. The above findings suggested that the antioxidant and immune-modulatory capacity of the abovementioned phytochemicals can improve gut function and health through multiple mechanisms [24]. However, further studies are warranted to uncover the detailed mechanisms that regulate gut function.

Serotonin (5-HT) is an important hormone and signaling molecule that regulates gut functions, including gut motility, ion transport, fluid secretion, and immune function [25,26,27]. More than 90% of 5-HT in the body is produced in the enterochromaffin cells (EC) of the gut through the metabolism of Trp by tryptophan hydroxylase 1 (Tph1). Extracellular 5-HT can be taken up by the serotonin reuptake transporter and then degraded to 5-hydroxyindoleacetic acid by monoamine oxidase A (Maoa) [28]. A study on the link between 5-HT and gut immune function showed that 5-HT altered colitis susceptibility in Tph1^−/−^ mice [29]. In addition, we have shown that Trp and *Limosilactobacillus mucosae* can alleviate dextran sulfate sodium (DSS)-induced colonic inflammation by upregulating the expression of 5-HT receptors (Htr), including *Htr1a* and *Htr4*, and reducing *Htr2a* and *Htr7* expression [30,31]. Moreover, a study in weaned piglets revealed that there was a positive correlation between *Htr7* expression and the levels of proinflammatory cytokines, including *IL-1β*, *IL-8*, and *TNF-α*, but a negative correlation between *Htr4* expression and levels of *IFN-γ* in the jejunum [32]. 5-HT was found to regulate intestinal motility via the activation of Htr3 and Htr4 [33]. In addition, microbial tryptamine stimulates gut transit by stimulating epithelial HTR4, which is accompanied by increased colonic fluid secretion in mice [34]. Therefore, the combination of 5-HT and Htr has a substantial effect on gut function [35]. However, the regulation of 5-HT metabolism and signaling by dietary bioactive compounds in the small intestine and the underlying mechanisms require further investigation.

This study aimed to identify the differences in the mode of action of different phytochemicals and Trp metabolites in the regulation of 5-HT signaling and ion transport in the mouse ileum using the Ussing chamber technique. The findings of this study will aid in the development of potential dietary interventions for improving gut health.

## 2. Results

### 2.1. Effects of Different Phytochemicals and Trp Metabolites on the Short-Circuit Current of the Mouse Ileum

**Figure 1:**
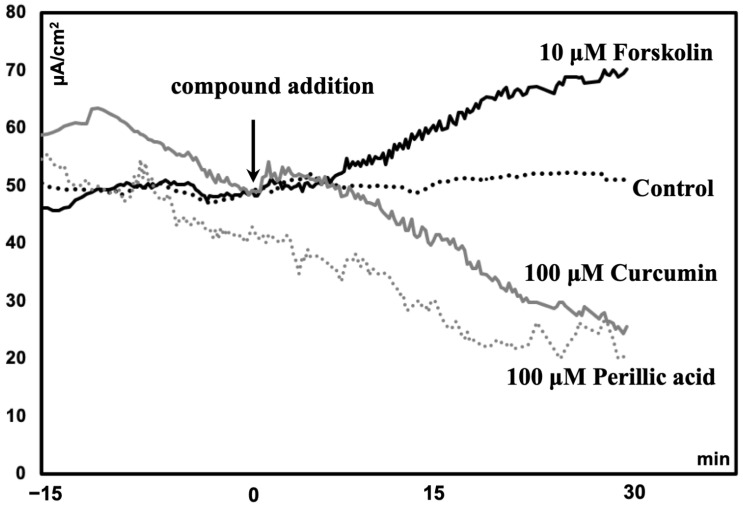
Representative diagram comparing the short-circuit current (Isc) of the ileum of mice following the administration of phytochemicals.

Compared to those in the control group, the ileum of mice treated with forskolin (10 μM) had greater Isc (*p* < 0.05). However, the VMA (100 μM) treatment group had a lower Isc (*p* = 0.05) than the control group (Table 1). Compared with the control, PA (100 μM) or curcumin (100 μM) tended to reduce Isc (*p* = 0.1) (Table 1). A representative diagram comparing the short-circuit current (Isc) following the administration of phytochemicals is shown in Figure 1.

### 2.2. Effects of Different Phytochemicals and Trp Metabolites on the Gene Expression and Protein Abundance of Key Enzymes Involved in 5-HT Metabolism in the Mouse Ileum

**Figure 2:**
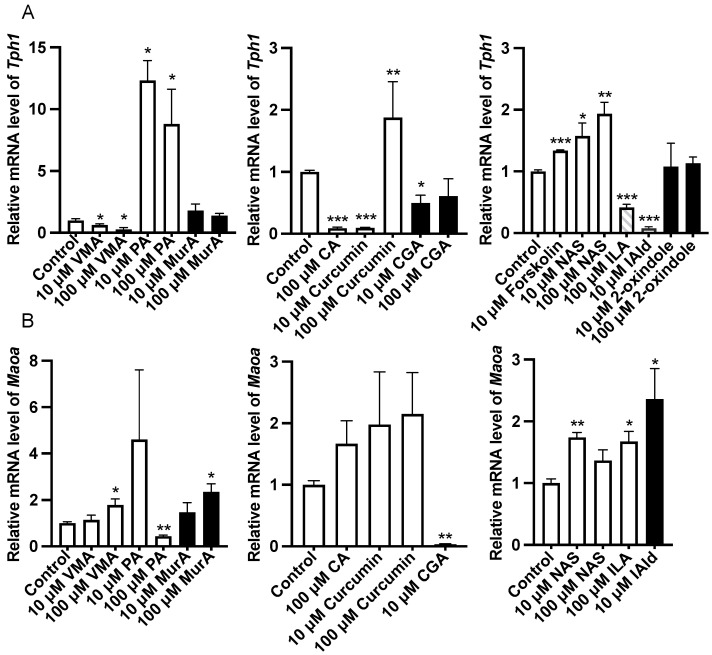
Effects of different phytochemicals and Trp metabolites on the gene expression of 5-HT metabolism-related enzymes, including *Tph1* (**A**) and *Maoa* (**B**) in the ileum of mice. The data in the charts are the means ± SEMs. * *p* < 0.05, ** *p* < 0.01, and *** *p* < 0.001 compared to the control group. Tph1, tryptophan hydroxylase 1; Maoa, monoamine oxidase A; VMA, vanillylmandelic acid; PA, perillic acid; MurA, muramic acid; CA, cinnamic acid; CGA, chlorogenic acid; NAS, *N*-acetylserotonin; ILA, indolelactic acid; IAld, indoleacetaldehyde. The number of animals is shown in Table 1.

**Figure 3:**
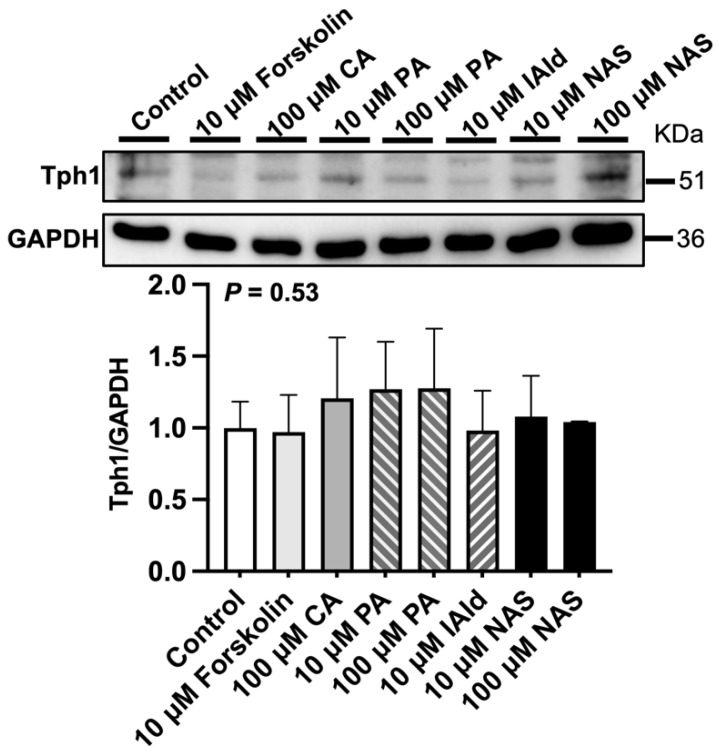
Effects of different phytochemicals and Trp metabolites on the protein abundance of tryptophan hydroxylase 1 (*Tph1*) in the ileum of mice. The data in the charts are the means ± SEMs, *n* = 3. CA, cinnamic acid; PA, perillic acid; IAld, indoleacetaldehyde; NAS, *N*-acetylserotonin.

Analysis of the gene expression of 5-HT metabolism-related enzymes in the ileum revealed that forskolin (10 μM), PA or NAS treatment upregulated (*p* < 0.05) the expression of *Tph1* compared with that in the control group (Figure 2). In addition, VMA, CA (100 μM), CGA (10 μM), ILA (100 μM), and IAld (10 μM) downregulated (*p* < 0.05) the expression of *Tph1* (Figure 2). The protein abundance of Tph1 in the ileum was not affected by the abovementioned phytochemicals and Trp metabolites (Figure 3). In addition, 100 μM VMA, MurA, ILA or 10 μM IAld upregulated (*p* < 0.05) the expression of *Maoa*. However, PA (100 μM) and CGA (10 μM) downregulated (*p* < 0.05) *Maoa* expression (Figure 2).

### 2.3. Effects of Different Phytochemicals and Trp Metabolites on the Gene Expression of Htr and Their Ratios in the Mouse Ileum

**Figure 4:**
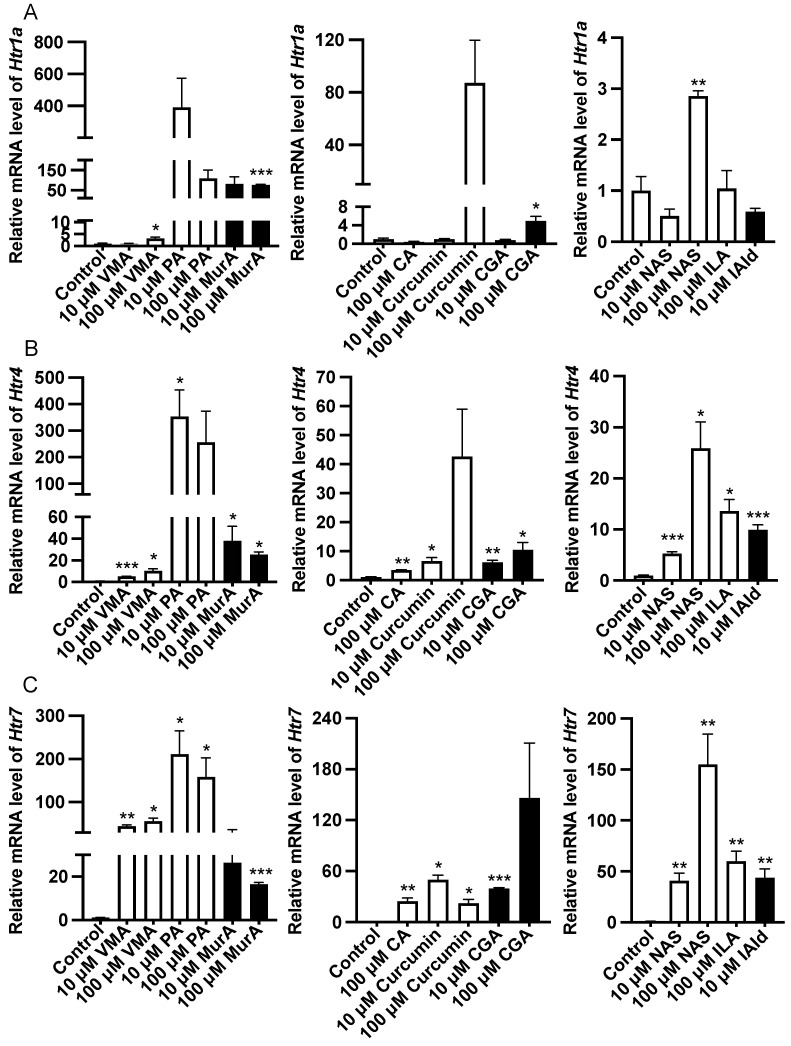
Effects of different phytochemicals and Trp metabolites on the expression of serotonin receptor genes, including *Htr1a* (**A**), *Htr4* (**B**), and *Htr7* (**C**) in the ileum of mice. The data in the charts are the means ± SEMs. * *p* < 0.05, ** *p* < 0.01, and *** *p* < 0.001 compared to the control group. The number of animals is shown in Table 1. VMA, vanillylmandelic acid; PA, perillic acid; MurA, muramic acid; CA, cinnamic acid; CGA, chlorogenic acid; NAS, N-acetylserotonin; ILA, indolelactic acid; IAld, indoleacetaldehyde.

Analysis of the gene expression of the Htr revealed that 100 μM VMA, GA, and NAS or 10 μM MurA upregulated (*p* < 0.05) the expression of *Htr1a* (Figure 4). Treatment with 10 μM VMA, PA, MurA, curcumin, CGA, NAS or IAld or 100 μM CA or ILA upregulated (*p* < 0.05) the expression of *Htr4* and *Htr7* (Figure 4).

**Figure 5:**
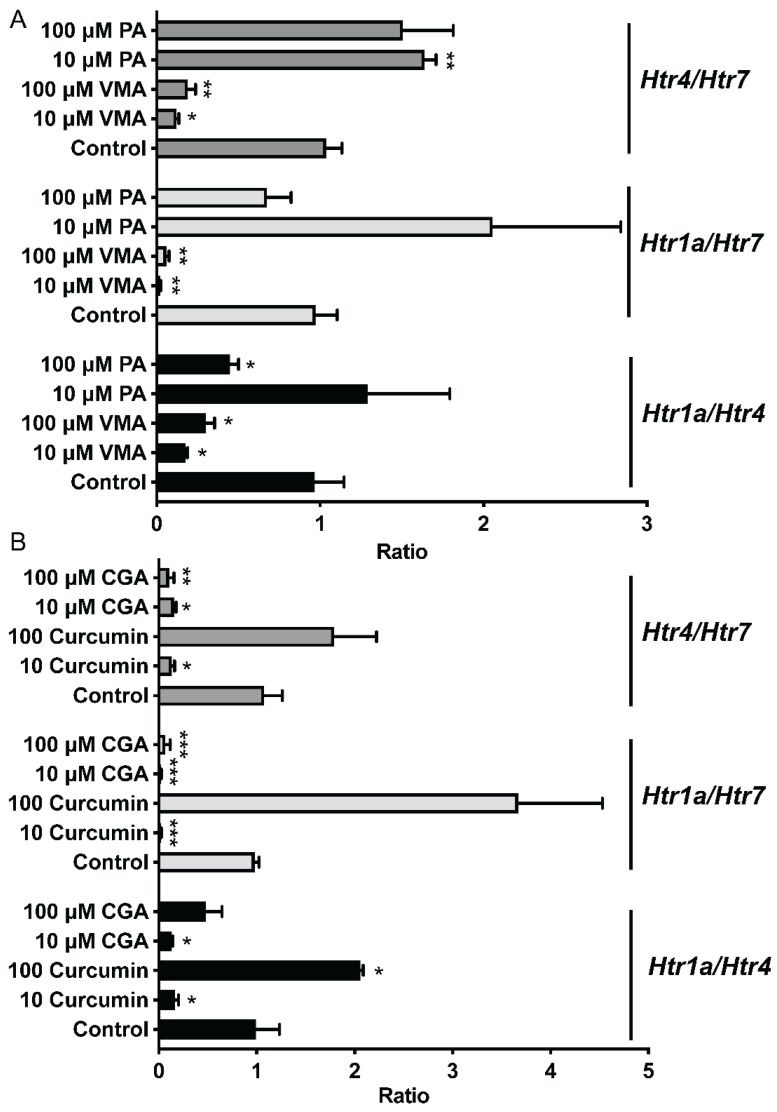
Different phytochemicals, including PA and VMA (**A**), and CGA and Curcumin (**B**) on the serotonin receptor gene ratios in the ileum of mice. The data in the charts are the means ± SEMs. * *p* < 0.05, ** *p* < 0.01, and *** *p* < 0.001 compared to the control group. The number of animals is shown in Table 1. CGA, chlorogenic acid; PA, perillic acid; VMA, vanillylmandelic acid.

To further compare the effects of selected phytochemicals and Trp metabolites on the expression of Htr, the expression ratios of *Htr1a*/*Htr7*, *Htr4*/*Htr7*, and *Htr4*/*Htr7* were calculated and analyzed based on their relative expression to that of *Gapdh* (Figure 5). Compared to those in the control group, the *Htr1a*/*Htr4* and *Htr4*/*Htr7* ratios in the curcumin (100 μM) and PA (10 μM) groups were greater (*p* < 0.05). However, the ileum treated with 10 μM or 100 μM VMA or CGA and 10 μM curcumin had lower (*p* < 0.05) *Htr1a*/*Htr7*, *Htr4*/*Htr7*, and *Htr1a*/*Htr4* ratios (Figure 5).

## 3. Discussion

Phytochemicals and Trp metabolites have been shown to improve gut function and homeostasis, and these health-promoting effects may be partially due to their regulation of gut 5-HT metabolism and signaling [35,36]. Gut barrier function is important not only for defense against luminal pathogens but also for nutrient absorption and electrolyte balance [37]. Our current study suggested that VMA reduced the short-circuit current, and 100 μM CGA and PA had a tendency to reduce the short-circuit current of the ileum. PA and NAS upregulated *Tph1* expression, while CA, CGA, ILA, or IAld downregulated *Tph1* expression. Moreover, PA or CGA downregulated *Maoa* expression. However, ILA and IAld upregulated *Maoa* expression. All selected phytochemicals and Trp metabolites upregulated the expression of *Htr4* and *Htr7*; however, PA increased the *Htr4*/*Htr7* ratio, while VMA and CGA reduced the *Htr1a*/*Htr7* and *Htr4*/*Htr7* ratios. These findings may help to gain further insight into the regulatory role of phytochemicals and Trp metabolites on gut 5-HT homeostasis-mediated gut function in health and disease.

The findings of our present study revealed that different Trp metabolites regulate gut 5-HT homeostasis differently. Mounting evidence has shown that the gut microbiota and its metabolites are crucial for the production of 5-HT by regulating *Tph1* expression in the intestines of rodents [38]. In particular, microbial-derived Trp metabolites exert diverse effects on the host physiology and gut function, including gut immune modulation [4,39]. Microbial indole derivatives such as ILA inhibit the proliferation of macrophages by reducing glycolysis, NF-κB, and hypoxia-inducible factor pathways and decreasing CCL2/7 in epithelial cells and mice [40]. In rat and mouse models, NAS can alleviate intestinal ischemic injury and DSS-induced chronic colitis [41,42,43]. In TNF-α-stimulated epithelial cells, the addition of ILA did not alter the expression of *Tph1* [44]. Additionally, microbial 5-hydroxyindole has been shown to promote 5-HT release from RIN14B cells, which regulates gut motility by activating Htr3 and Htr4 [39]. In our study, the Trp metabolite NAS upregulated the expression of *Tph1*, while ILA and IAld downregulated the expression of *Tph1*, suggesting that the regulation of 5-HT production differs among different Trp metabolites. Notably, the abovementioned regulatory role of different Trp metabolites may also be affected by different physiological conditions of the gut (i.e., normal conditions vs. inflammation) [44]. Further studies are required to determine the differential effects of various Trp metabolites on 5-HT signaling to identify new targets for dietary strategies to improve gut function.

Our current study also reinforces the importance of gut 5-HT metabolism and signaling in the anti-inflammatory effects of phytochemicals. The limonene derivative PA has been widely used in food, medicine, and cosmetics [13]. PA was proposed to be a potential anticancer, immunomodulatory, and anti-obesogenic bioactive compound [45]. However, there are very few studies on the regulatory role of PA on gut 5-HT. In mice, PA alleviated radiation-induced small intestinal histopathological damage and reduced the production of the proinflammatory cytokines IL-1β and TNF-α [15]. In addition, supplementation with 0.1% D-limonene hampered diet-induced obesity in mice with increased levels of PA and perillic acid-8,9-diol in the urine [46]. Our current results demonstrated that PA stimulated 5-HT synthesis, inhibited 5-HT degradation, and upregulated *Htr4* and *Htr7* expression. We therefore deduced that PA might exert its beneficial effects by affecting 5-HT metabolism and Htr, but this needs to be further confirmed in vivo. Additionally, the present findings confirmed that CGA downregulated the expression of *Tph1* and *Maoa*, which is in line with a previous finding that CGA suppressed Maoa activity and prevented 5-HT from being deaminated [47]. In addition, studies have shown that curcumin can modulate 5-HT levels in the brain and exert antidepressant and anticonvulsive effects [48,49,50]. Additionally, curcumin has been widely reported to promote intestinal health by affecting cross-talk among cell signaling pathways, immune function, and the gut microbiota [24,51]. However, a recent study suggested that higher concentrations (20–80 μM) of curcumin exerted cytotoxic effects on human small intestine epithelial cells, which might be a compensatory protective mechanism under conditions of impaired cell vitality [52]. Notably, our current results revealed that curcumin at low and high concentrations had opposite effects on *Tph1* expression in the ileum, indicating that its effect on 5-HT signaling may be dose dependent. Moreover, the levels of 5-HT were decreased in the colon of rats with irritable bowel syndrome by curcumin supplementation, but an Htr1a antagonist reversed this effect, indicating that the beneficial effect of curcumin is Htr1a dependent [53]. This finding is in line with our current finding that the *Htr1a*/*Htr4* ratio was increased by 100 μM curcumin. Taken together, the intricate role of 5-HT and its receptors in the regulation of gut function modulated by the abovementioned compounds requires further investigation.

The expression of different subtypes of Htr in the gut plays vital roles in gut functions, including gut secretion, motility, and immune modulation [33]. Despite a great clinical interest and an increasing number of studies devoted to Htr, the mechanism modulating Htr effect on physiological and pathological functions required further validation. A previous study showed that the Htr1 and Htr7 receptors can form homo- and heterodimers [54], thus impairing the ability of Htr1 to activate the Gi-protein system and further downstream signaling pathway. Especially, a recent report found that overexpression of Htr7 led to the decreased abundance of Htr1a in the membrane protein fraction from the midbrain samples of C57BL/6 mice [55], thus exerting an antidepressive effect, which suggests the interaction and proportion of Htr played important roles in the physiology and pathology of the body. However, changes in the proportion of Htr in the gut and the effects and mechanisms of Htr interaction on gut function in gut health and disease remain poorly understood. A recent study in weaned piglets indicated that there is a correlation between the expression of Htr, including *Htr4* and *Htr7*, and the production of inflammatory factors in the jejunum [32]. In addition, Trp upregulated *Htr1a* and *Htr4* expression, thus mitigating DSS-induced colitis in mice [30]. Interestingly, although the inhibition of Htr1a or Htr4 by their antagonists exacerbated DSS-induced colitis in mice, the modulation of one Htr via its antagonist did not change the expression of other Htr [30]. In our current study, CGA stimulated the expression of *Htr1a*, *Htr4*, and *Htr7*, but the *Htr1a*/*Htr4*, *Htr1a*/*Htr7*, and *Htr4*/*Htr7* ratios were lower than those in the control group. Hence, we speculated that the relative proportion of each Htr in the intestine may be vital for the regulation of gut homeostasis; however, more studies are warranted to test this hypothesis.

The Ussing chamber system has been extensively used for evaluating the transport of ions, nutrients, and drugs across various epithelial tissues, as well as intestinal permeability [56]. Short-circuit current (Isc) is an indicator of the tissue’s ability to absorb or secrete [57]. Our results indicated that 10 μM forskolin increases Isc significantly, and this suggested the ion transport through ileum epithelium tissue was active. However, a higher dose (100 μM) of PA and curcumin tended to decrease the Isc in the ileum of the mice, which would suggest either an inherent change in epithelial transport or a decrease in fluid secretion. In addition, activation of ion transport in the intestine can be achieved in part through the production of hormones from enteroendocrine cells. Hormones, such as 5-HT, can in turn act on mucosal nerve endings and activate secretomotor neurons by activating Htr [58,59]. Therefore, whether PA-induced Isc change is caused by the indirect 5-HT signaling effect can be explored by the addition of selective blockers of Tph1 responsible for 5-HT synthesis or Htr antagonists in future studies. Also, one limitation of our present study is that the sample size is relatively small, so more sample sizes need to be replicated in future experiments to verify the above speculation. In addition, by using an EC-enriched monolayer system, scholars found that treatment with forskolin (10 μM) and the dietary nutrient curcumin (100 μM) stimulated 5-HT production by cells [60]. This result was partly consistent with our finding that Isc and *Tph1* gene expression were elevated by forskolin. However, the findings regarding the ability of curcumin to stimulate 5-HT release were inconsistent with our experimental results, as evidenced by the fact that 100 μM curcumin treatment reduced Isc and *Tph1* expression in our study. This might be due to the differences in the experimental systems used in the two studies [60].

## 4. Materials and Methods

### 4.1. Reagents

Primary antibody against Tph1 (Cat# 12339) was purchased from Cell Signaling Technology (Danvers, MA, USA). Glucose and salts were purchased from Sangon Biotech Co., Ltd. (Shanghai, China). Curcumin (#HY-N0005), forskolin (#HY-15371), indolelactic acid (ILA, #HY-113099), and 2-oxindole (#HY-Y0061) were obtained from Med Chem Express Co., Ltd. (Shanghai, China), and the purity of these reagents is more than 98%. Cinnamic acid (CA, #C80857), chlorogenic acid (CGA, #C3878), indoleacetaldehyde (IAld, #I1000), muramic acid (MurA, #M2503), *N*-acetylserotonin (NAS, #A1824), perillic acid (PA, #218359), and vanillylmandelic acid (VMA, #H0131) were purchased from Merck Sigma-Aldrich (Shanghai, China), and the purity is more than 95%. The compounds used in this study were dissolved in dimethyl sulfoxide (DMSO, final concentration 0.1%) and further diluted with Krebs’ solution for subsequent Ussing chamber experiments. The concentrations of the substances were selected with reference to the possible non-toxic dosages that have been reported in cell and mice experiments. Chemical structures and certain physicochemical properties of the above compounds used in this study are shown in Table 2.

### 4.2. Animals

In this study, sixty 10~12 week-old C57BL/6 male mice with an average body weight of 27.0~30.0 g were purchased from Beijing HFK Bioscience (Beijing, China). Upon arrival, the mice were allowed to acclimate for 7 days before the experiment. The mice were housed in polycarbonate cages in a specific-pathogen-free environment and were kept at 22–25 °C and 45–55% relative humidity with a 12 h light/dark cycle and ad libitum water and feed. The standard rodent diet (Cat#1032, Beijing HFK Bioscience) used in the experiments was the same as that used in our previous study [30].

### 4.3. Ussing Chamber Experiments

Before Ussing chamber analysis, the mice were anesthetized with ether before they were euthanized by cervical dislocation, according to the previous method [31]. The proximal ileum was first rinsed with precooled Krebs’ solution (11.1 mM glucose, 118 mM NaCl, 4.8 mM KCl, 1.0 mM NaH_2_PO_4_, 1.2 mM MgSO_4_, 25 mM NaHCO_3_, 2.5 mM CaCl_2_, pH = 7.4) to remove the chyme and then opened along the mesenteric border.

**Figure 6:**
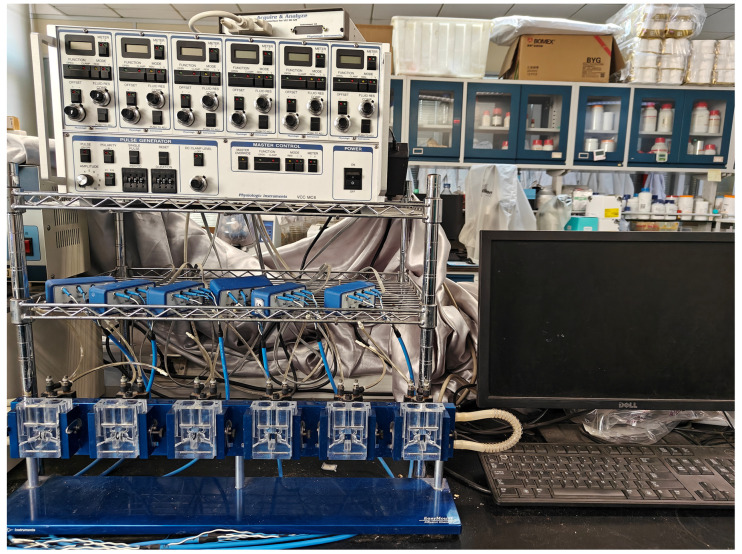
A photo of the Ussing chamber system used in this study.

A six-channel Ussing chamber system (MC6-6, Physiologic Instrument, Reno, NV, USA) was used to measure the short-circuit current (Isc) (as shown in Figure 6). The isolated ileum segments of 1.5 cm in length were then pinned onto Ussing chamber sliders (P2300, 0.2 cm^2^ apertures) within 15 min after sampling. After that, the sliders with the ileum were mounted into chambers (EasyMount Diffusion Chambers, Physiologic Instruments), and 5 mL of Krebs’ solution was added to the mucosal side and serosal side of the ileum. A final concentration of 5 mM mannitol was added to the mucosal side to limit active transport while maintaining osmotic balance. Ileal tissue was stabilized for 15 min before clamping the voltage to 0 V before treatment. Final concentrations of 10 μM or 100 μM of the selected compounds were added to the chamber for up to 40 min after the mounted tissues were stabilized. Forskolin (10 μM) was used as a positive control, as reported previously [61]. In this study, the number of ileal tissues tested used for each compound was equal to the number of animals used (*n* = 3~6). The Isc was continuously recorded by Acquire & Analyze software 2.3 (Physiologic Instruments, Reno, NV, USA). Tissue viability was assessed according to a previous method [61], and tissues with a <1 mV increase in the transepithelial potential difference were excluded from further analysis.

### 4.4. RNA Extraction and Quantitative Real-Time PCR Analysis

After Ussing chamber analysis, total RNA was extracted from the analyzed ileal tissues using an RNAiso Plus kit (Takara, Beijing, China). The integrity and purity of the RNA were determined using a Nanodrop P330 (Implen, Munich, Germany) and electrophoresis. RNA was then reverse-transcribed using a FastKing RT kit from TIANGEN Biotech Co., Ltd. (Beijing, China). Quantitative real-time PCR was performed using a SYBR Premix Ex Taq II (Takara, Beijing, China) with an ABI 7500 real-time PCR detection system (Thermo Fisher, Waltham, MA, USA). The sequences of the primers (*Tph1*, *Maoa*, *Htr1a*, *Htr4*, *Htr7*, and *GAPDH*) used for this study were described previously [30]. GAPDH was utilized as the internal reference. The 2^−ΔΔCt^ method was used for the quantification of gene expression.

### 4.5. Western Blot Analysis

Ileum samples were homogenized in liquid nitrogen, and protein was extracted and measured for the abundance of Tph1 by Western blot analysis, according to a previous protocol [62]. Protein bands were measured via ECL Plus detection reagents (Thermo Fisher, Waltham, MA, USA) and visualized by the Image Quant LAS 4000 mini system (GE Healthcare, Piscataway, NJ, USA). The band intensity of each target protein was compared with that of GAPDH by Image J software version 1.53 (NIH, Bethesda, MD, USA).

### 4.6. Statistical Analysis

The values are presented as the means ± SEMs. Statistical differences between two groups were analyzed by t-tests. The data were assessed by one-way ANOVA followed by Duncan’s multiple comparison test. SPASS 22.0 (IBM, Armonk, NY, USA) and Prism 9.0 (GraphPad, Boston, MA, USA) were used for statistical analysis. *p* < 0.05 was used to indicate a significant difference.

## 5. Conclusions

In conclusion, 100 μM VMA or curcumin reduced Isc, and PA and NAS upregulated the gene expression of *Tph1*, but CGA (10 μM), IAld (10 μM), and ILA (10 μM) downregulated the gene expression of *Tph1*. The selected phytochemicals and Trp metabolites upregulated the expression of *Htr4* and *Htr7* compared to those in the control group. PA (10 μM) increased the *Htr4*/*Htr7* ratio, while CGA decreased the *Htr1a*/*Htr4*, *Htr1a*/*Htr7*, and *Htr4*/*Htr7* ratios. These findings may help to elucidate the regulatory role of phytochemicals and Trp metabolites in gut 5-HT signaling-mediated gut ion transport and homeostasis, thereby providing potential dietary intervention options for improving gut function and health in humans and animals.

## Figures and Tables

**Table 1:** Effects of different phytochemicals and Trp metabolites on the short-circuit current (Isc) across the mouse ileum ^1^.

Components	Concentration (μM)			*p* Value
	0	10	100	
Forskolin	1.00 ± 0.11	1.68 ± 0.20	ND	<0.05
Phytochemicals				
CA	1.00 ± 0.14	ND	0.81 ± 0.08	0.52
CGA	1.00 ± 0.01	0.99 ± 0.02	0.87 ± 0.01	0.12
Curcumin	1.00± 0.03	1.05 ± 0.36	0.76 ± 0.08	0.19
MurA	1.00 ± 0.18	0.85 ± 0.14	1.07 ± 0.36	0.51
PA	1.00 ± 0.13	0.76 ± 0.17	0.63 ± 0.12	0.14
VMA	1.00 ± 0.15 ^a^	0.57 ± 0.10 ^ab^	0.41 ± 0.01 ^b^	0.05
Trp metabolites				
IAld	1.00 ± 0.10	0.97 ± 0.08	ND	0.85
ILA	1.00 ± 0.05	ND	0.93 ± 0.24	0.79
NAS	1.00 ± 0.24	0.97 ± 0.23	1.06 ± 0.25	0.80
2-Oxindole	1.00 ± 0.09	0.99 ± 0.14	1.02 ± 0.04	0.90

^1^ The values in the table are the means with SEM. Labeled means in a row without a common letter differ, *p* < 0.05. CA, cinnamic acid; CGA, chlorogenic acid; MurA, muramic acid; PA, perillic acid; VMA, vanillylmandelic acid; Trp, tryptophan; IAld, indoleacetaldehyde; ILA, indolelactic acid; NAS, *N*-acetylserotonin; ND, not determined. Forskolin: *n* = 4; CA: *n* = 6; CGA: (10 μM) (*n* = 3) and (100 μM) (*n* = 3); Curcumin: (10 μM) (*n* = 3) and (100 μM) (*n* = 6); MurA: (10 μM) (*n* = 4) and (100 μM) (*n* = 3); PA: (10 μM) (*n* = 3) and (100 μM) (*n* = 4); VMA: (10 μM) (*n* = 4) and (100 μM) (*n* = 3); IAld: *n* = 5; ILA: *n* = 4; NAS: (10 μM) (*n* = 3) and (100 μM) (*n* = 3); 2-Oxindole: (10 μM) (*n* = 3) and (100 μM) (*n* = 3).

**Table 2:** Chemical structures and certain physicochemical properties of the selected compounds used in this study.

Components	Molecular Structure	Molecular Formula	Molecular Weight (g/mol)	LogP	Melting Point (°C)
Forskolin	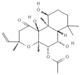	C_22_H_34_O_7_	410.5	1	230~232
Phytochemicals					
CA	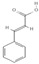	C_9_H_8_O_2_	148.16	2.13	133
CGA	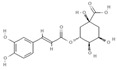	C_16_H_18_O_9_	354.31	− 0.4	205~209
Curcumin	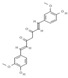	C_21_H_20_O_6_	368.4	3.2	183
MurA	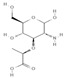	C_9_H_17_NO_7_	251.23	− 4.6	153
PA	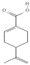	C_10_H_14_O_2_	166.22	1.4	60
VMA	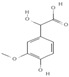	C_9_H_10_O_5_	198.17	− 0.2	132~134
Trp metabolites					
IAld	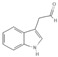	C_10_H_9_NO	159.18	1.3	N/A
ILA	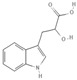	C_11_H_11_NO_3_	205.21	1.5	145~146
NAS	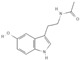	C_12_H_14_N_2_O_2_	218.25	0.5	120~122
2-Oxindole	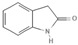	C_8_H_7_NO	133.15	1.2	123~128

CA, cinnamic acid; CGA, chlorogenic acid; MurA, muramic acid; PA, perillic acid; VMA, vanillylmandelic acid; Trp, tryptophan; IAld, indoleacetaldehyde; ILA, indolelactic acid; NAS, *N*-acetylserotonin; N/A, not applicable.

## Data Availability

All data presented in the study were included in the manuscript.
